# Experiences at recovery community centers predict holistic recovery outcomes: a daily diary assessment of RCC helpfulness, meaningfulness, and recovery identity

**DOI:** 10.3389/fpubh.2024.1476441

**Published:** 2025-01-14

**Authors:** Hannah B. Apsley, Joseph Lancaster, Wen Ren, Timothy Brick, H. H. Cleveland

**Affiliations:** ^1^Department of Human Development and Family Studies, Pennsylvania State University, University Park, PA, United States; ^2^Institute for Computational and Data Sciences, Pennsylvania State University, University Park, PA, United States

**Keywords:** addiction recovery, recovery community center, helpfulness, meaningfulness, recovery identity, daily diary

## Abstract

**Introduction:**

Recovery community centers (RCCs) offer various support services to people in addiction recovery, such as hosting mutual help meetings and sober social activities and providing employment support and recovery coaching. To date, very little is known about RCCs and their relationship with recovery outcomes, as well as how RCCs may vary in helpfulness from visit to visit. This study used a daily diary approach to assess the intraindividual variation of daily RCC helpfulness, and whether RCC helpfulness predicted the holistic recovery indices of daily meaningfulness and recovery identity.

**Methods and materials:**

RCC attendees (analytical *N* = 88) from RCCs in Pennsylvania completed daily diary assessments using a smartphone application, for 10 consecutive days. If participants reported that they had spent time at the RCC that day, they then reported the perceived helpfulness of the RCC visit using 7 items. Participants also reported their daily meaningfulness and recovery identity. Ultimately, participants visited their RCC on 247/799 (30.9%) of all reported study days. Multilevel models were used to assess the hypotheses.

**Results:**

Participants generally reported that their RCC visits were very helpful (M = 87.13 [scale of 0–100], SD = 13.26). Nearly half of the variation in RCC helpfulness was attributable to intraindividual variation (ICC = 0.51). Multilevel models revealed that both interindividual and intraindividual RCC experiences predicted increased holistic recovery outcomes, over the prior day. Individuals’ mean levels of perceived RCC helpfulness, as well as person-mean-centered RCC daily helpfulness, positively predicted daily meaningfulness and recovery identity.

**Conclusion:**

RCCs predict the holistic recovery outcomes of meaningfulness and recovery identity outcomes on the particular days that the RCCs are visited, and for the individuals who find RCCs more helpful overall. This study offers preliminary evidence to suggest that RCCs are appropriate recipients of public funding intended to support recovery in US communities.

## 1 Introduction

This paper addresses three specific calls to action by addiction recovery researchers. The first is that recovery studies should recruit from, and assess the efficacy of, non-professional recovery support settings (1, 2), as these are utilized almost as frequently as professional treatment services (3). The second is that research on recovery ought to shift its focus from relapse and consumption-related outcomes to a focus on outcomes indicative of general wellbeing, health and functioning (1, 4, 5). Third, because recovery is a dynamic and individualized process, recovery research should employ methods that are appropriate to capture dynamic experiences (1, 6). This study meets these calls to action by assessing the daily relationship between the quality of experiences at a setting for recovery support services, and non-consumption indices of recovery.

Recovery support services aim to help those in recovery move from recovery initiation to sustained recovery long-term (2), and are utilized by an estimated 21.8% of American adults who have a resolved past substance use concern (3). These services are often peer-led, and can include sober housing, recovery coaching, faith-based recovery services, and practical support such as transportation to recovery-related appointments (2, 3). In the dynamic behavioral ecological model of recovery, recovery support services settings are categorized as “neighborhood and built environments supportive of recovery,” and interact with mutable individual factors such as one’s access to social support (6). Indeed, the general model of these support services is that they support recovery by facilitating access to recovery capital, such as social capital in the form of supportive friendships and mentors, and physical capital in the form of shelter or transportation (2). Having access to these supports is important for those millions of people in recovery who cannot—or do not want to—access professional treatment, and because even among those who participate in treatment, an estimated 40–60% of patients return to active substance use within a year (7). This implies that professional treatment is often not sufficient to build recovery long-term.

One of the settings for recovery support services is recovery community centers (RCCs). Although there is variation from center to center, RCCs are generally drop-in centers run by volunteers (8), and facilitate formal services such as organized mutual help meetings and recovery coaching (8–10), as well as informal supports such as socializing with recovery peers and connecting to the recovery community (31). At the cross-sectional level, attendance at these centers has been associated with positive recovery outcomes such as reduced substance use (11, 12) and psychological distress (13), and improved quality of life and self-esteem (13). At the daily level RCC visits have been associated with lower substance craving and negative affect (14). However, it is highly likely that RCC visits differ from one another in their quality and helpfulness. Prior work has indicated that there is a high degree of intraindividual variation in the activities that attendees engage in from visit to visit (31), and that attendees rate some RCC activities as more helpful than others, on average (13). For instance, participants may visit the RCC on some days to access the internet or other technology provided there, which has been rated as very helpful on average by RCC participants (13). On other days, attendees may visit the RCC to receive financial services, which RCC participants have rated as comparatively less helpful (13).

Ashford and colleagues define recovery from substance use disorder (SUD) as “an individualized, intentional, dynamic, and relational process involving sustained efforts to improve wellness” [(15), p. 183]. Other definitions of recovery detail examples of holistic wellness outcomes, such as physical and mental health (16), improved quality of life (16–18), social relationships or support (16, 17) and a sense of purpose (4). Kelly and Hoeppner’s (18) biaxial formulation of recovery (2015) posits that these constructs are important not only as outcomes, but because they also in turn behave as forms of recovery capital which then bolster further recovery. Cleveland (1) and Wikiewitz and Tucker (5) have both recently highlighted that more research on these holistic outcomes is needed in varying recovery contexts. Having a greater understanding of how these positive holistic outcomes emerge and are sustained within these contexts will both enrich theory and provide useful information for those who work in recovery support services.

This paper focuses on two specific holistic indices of recovery. The first is a general sense of meaningfulness in daily life. Meaningfulness is the “set of subjective judgements people make that their lives are (a) worthwhile and significant, (b) comprehensible and make sense, and (c) marked by the embrace or pursuit of one or more highly valued, overarching purposes or missions” [(19), p. 961]. Meaningfulness, and engagement in meaningful activities such as work, education and volunteering, have been cross-sectionally associated with self-worth, pride (20) and wellbeing (21), and longitudinally associated with better health and overall quality of life (22), for people in recovery. Because many RCCs offer participants opportunities to engage in meaningful activities, such as volunteering, and supporting their peers in recovery, we anticipate that engagement with RCCs will predict a daily sense of meaningfulness.

The second index of recovery is recovery identity. Recovery identity refers to the degree to which an individual identifies with a social group of those who are also in recovery and adheres to their behavioral norms (23). Identifying as a person in recovery is in contrast with having an identity as someone who uses substances (23, 24). Recovery identity has been associated with higher self-efficacy, lower relapse rates (23) and environmental quality of life (25). It is likely that associating with an RCC will foster recovery identity, because RCCs aim to facilitate strong and supportive social relationships with others in recovery.

As detailed above, individuals’ experiences of RCC visits are likely to vary from visit to visit, particularly in the activities that participants engage in while there (31). Variability has also been documented for aspects of recovery examined herein. For example, in a sample of patients in inpatient treatment for opioid use disorder, there was a moderately high degree of intraindividual variation in daily meaningfulness, and this daily meaningfulness was predicted by positive social experiences (26). In non-recovery samples, different aspects of personal identity have exhibited variation from day to day (27, 28), and we anticipate that this will also be the case for recovery identity, specifically. Because both RCC experiences, and indices of recovery are dynamic, this study employs a daily diary approach; daily diary designs have been identified as a strategy which meets the minimum requirements for assessing the dynamic interactions between recovery contexts (such as RCCs) and mutable individual recovery factors (6). Daily diary assessments also have the advantage of reducing retrospective bias and state congruent recall, as well as allowing for the estimation of both inter and intraindividual variation (29).

This study investigates one preliminary research question, and two specific hypotheses:

**Preliminary RQ:** To what degree does RCC’s attendees’ perception of the helpfulness of their RCC vary across visits?

*Hypothesis*: Perceived helpfulness of visits to RCCs will predict holistic indices of recovery at the daily level.

*H1*: Daily RCC helpfulness will predict greater meaningfulness compared to the prior day.

*H2*: Daily RCC helpfulness will predict greater recovery identity compared to the prior day.

## 2 Materials and methods

### 2.1 Recruitment and procedures

In 2022 and 2023, participants (*N* = 94) were recruited from 6 different RCCs across the state of Pennsylvania. These participants were recruited in partnership with RCC leaders and staff members, who distributed fliers and emails regarding the study. Study team members met with interested individuals as a group during in-person recruiting meetings held at the RCCs. After receiving a description of study procedures, the survey app, and consent procedures, RCC members were asked to join the study. Individuals who chose to join the study then took part in the consent procedure, and completed a paper and pen baseline survey. The baseline survey included demographic items, and items about substance use history and past treatment and recovery experiences.

After completing the baseline survey, participants downloaded the PSU Wear-IT smartphone application (32). For 10 days, the app sent participants a push notification at 8:30 pm indicating that a new survey was available. The app also sent reminder notifications every 30 min for 2 h. Surveys took roughly 10 min to complete. Participants received $10 for completing the baseline survey, and $6 for each daily survey completed, both in the form of an electronic gift card following study completion. All study procedures were approved by the Pennsylvania State University Institutional Review Board.

### 2.2 Measures

#### 2.2.1 RCC helpfulness

Each day, participants reported whether they had spent any time at an RCC. If they responded affirmatively, they were then presented with seven items regarding how helpful or supportive their experience at the RCC was that day, with the stem *When thinking about your time at the RCC today…*. Example items included *How helpful to your recovery was being at the RCC?* and *How supported did you feel by other members and staff at the RCC?* Participants rated these items using a sliding scale of 1–100, with the labeled anchors of *not at all* (0) to *very* (100). The scale was calculated by averaging the seven items.

These items have not previously been published and were developed by the study team for this project. All seven items, along with their descriptive statistics are provided in Supplementary Table S1. Cronbach’s Alpha for this scale, across all days and participants is 0.95. To assess the variability of responses across participants, study days, and the seven items, an ANOVA with random effects (i.e., intercept-only model) was fit to the items. This ANOVA calculated variance components that were then used to calculate a reliability coefficient [R_c_ (30);]. The reliability coefficient for these seven items is 0.91, meaning that these items reliably measure within-person change of RCC quality across days.

In preparation for the multilevel models, means were calculated for each participant to create a between-person variable of each person’s average perception of RCC quality across study days. These means were subtracted from each individual observation, to create a person-mean centered daily variable.

#### 2.2.2 Outcomes

Daily meaningfulness was measured using five items, including *My day has been meaningful* and *My day has been gratifying.* Participants rated their agreement with these items using a sliding scale of 1–100, with the labeled anchors of *strongly disagree* (0) to *strongly agree* (100). The scale was calculated by averaging the five items. This scale has been previously used with a sample of participants in treatment for SUD (26). This scale has high indices of reliability (*α* = 0.96; R_c_ = 0.93).

Daily recovery identity was measured using nine items with the stem “Thinking about today, I feel like…” Items included *I kept my recovery central to my day* and *I was grateful to be in recovery*. Participants rated their agreement with these items using a sliding scale of 1–100 with the labeled anchors of *strongly disagree* (0) to *strongly agree* (100). One item, regarding missing one’s old drug use/drinking social group, was reverse-scored such that higher scores indicated having a stronger recovery identity that day. The scale was calculated by averaging the nine items. This scale was recently utilized for this sample by Lancaster et al. (14). This scale has high indices of reliability (*α* = 0.91; R_c_ = 0.86).

The means and other descriptions of the study variables are detailed in Table 1.

**Table 1:** Description of study variables.

	*M*	SD	Range	ICC	*α*	R*_c_*
Personal RCC helpfulness	87.13	13.26	45–100	-	-	-
Daily RCC helpfulness (Raw)	87.05	14.67	29.86–100	0.51	0.91	0.91
Daily meaningfulness	78.50	21.5	0–100	0.40	0.96	0.93
Daily recovery identity	82.47	16.92	1.78–100	0.57	0.91	0.86

### 2.3 Analytical strategy

The preliminary research question was assessed by calculating the intraclass correlation (ICC) for daily RCC helpfulness. The ICC was calculated by first, running a multilevel model in which RCC helpfulness was predicted using only random slopes, using the *lmer* function in the *lme4* package in R. The amount of variance in the intercept (or interindividual variation) was then divided by the total amount of variance in the model, using the *icc* function in the *mlmhelpr* package. The ICC represents the proportion of the total variance that is attributable to between-person differences in RCC helpfulness.

The hypotheses were addressed using four different multilevel models, two for each of the respective outcomes (daily meaningfulness, and daily recovery identity). The first model for each outcome predicted the daily outcome using: a random intercept, the person-mean centered value of RCC experience quality that day, and each individual’s mean level of RCC experience quality across the study period. These models include random intercepts, but not random slopes. The authors explored including random slopes in the models, but the limited number of RCC visit days led to both convergence and singularity issues. The multilevel equation for these models is as follows:

Level 1 (within-person):


$$y_{\mathrm{id}}=\beta_{0i}+\beta_{1}RCC\_\mathrm{Helpful}\_\mathrm{pm}c_{\mathrm{id}}+\varepsilon_{\mathrm{id}}$$

Level 2 (between-people):


$$\beta_{0i}=\gamma_{00}+\gamma_{01}RCC\_\mathrm{Helpful}\_\mathrm{persona}l_{i}+u_{0i}$$

$$\beta_{1}=\gamma_{1}$$

At level 1, $y_{\mathrm{id}}$ indicates the outcome (either recovery identity, or meaningfulness) for each specific person (*i*), on each day (*d*). This variable was regressed onto the intercept for each individual ( $\beta_{0i}$ ), the slope ( $\beta_{1}$ ) of person-mean centered RCC helpfulness for each person on each day ( $RCC\_\mathrm{Helpful}\_\mathrm{pm}c_{\mathrm{id}}$ ). $\varepsilon_{\mathrm{id}}$ represents the residual value for each individual on each day (i.e., level 1). At Level 2, individual intercepts ( $\beta_{0i}$ ) were comprised of the overall intercept for all participants ( $\gamma_{00}$ ), the slope ( $\gamma_{01}$ ) for personal mean levels of RCC helpfulness for each person ( $RCC\_\mathrm{Helpful}\_\mathrm{persona}l_{i}$ ), and residuals for each individual ( $u_{0i}$ ) between the overall intercept, and individual intercepts.

The second model for each outcome included an additional predictor. This additional variable was the prior-day outcome, to approximate directionality. By including the prior-day outcome in the model as a predictor, any statistically significant relationship between the predictors and the outcome controlled for the level of the outcome from the prior day. This inclusion addresses the possibility that a significant same-day relationship between predictors and the outcome may be due to individuals having more helpful RCC experiences on given days due to the prior days’ relatively high (for that individual) recovery identity or meaningfulness (rather than vice versa). The equation for these two models is as follows:

Level 1 (within-person):


$$y_{\mathrm{id}}=\beta_{0i}+\beta_{1}RCC\_\mathrm{Helpful}\_\mathrm{pm}c_{\mathrm{id}}+\beta_{2}y_{\mathrm{id}-1}+\varepsilon_{\mathrm{id}}$$

Level 2 (between-people):


$$\beta_{0i}=\gamma_{00}+\gamma_{01}RCC\_\mathrm{Helpful}\_\mathrm{persona}l_{i}+u_{0i}$$

$$\beta_{1}=\gamma_{1}$$

$$\beta_{2}=\gamma_{2}$$

All variables in these models remain unchanged from previous models, with $y_{\mathrm{id}-1}$ representing the previous day outcome for individual i and $\beta_{2}$ representing the slope of the previous day outcome. The authors anticipated that this second set of analyses would include relatively few observations. This is because these analyses exclude (1) any day that the RCC wasn’t visited, (2) each participant’s first study day (as these days inherently have no lagged day), and (3) any day that participants did not report on consecutive study days. Because of this limited number of observations, the authors anticipated that there might be concerns with power and hence, opted to include models both with and without a lagged-day autoregressive predictor to assess the research questions.

## 3 Results

### 3.1 Participants, RCC visits, and helpfulness ICC

RCCs were visited on 30.9% of all reported study days (247/799 days). 6 participants (6.4%) never visited the RCC, and were excluded from the analyses. A description of the 88 participants in the analytical sample is detailed in Table 2. Daily RCC helpfulness had an ICC of 0.51.

**Table 2:** Demographic characteristics of the sample (*N* = 88).

	*M* (SD)/*N* (%)
Age in Years	42.81 (12.49)
Sex-Female^†^	49 (55.7%)
Race	
American Indian/Alaska Native	1 (1.1%)
Black/African American	14 (15.9%)
White	69 (78.4%)
Multiracial	3 (3.4%)
Other	1 (1.1%)
Hispanic ethnicity	1 (1.1%)
Employment status	
Unemployed	27 (30.7%)
Part-Time	14 (15.9%)
Full-Time	41 (46.6%)
Retired	2 (2.3%)
Annual household income	
Less than $10,000	17 (19.3%)
$10,000 to $24,999	23 (26.1%)
$25,000 to $49,999	20 (22.7%)
$50,000 to $74,999	11 (12.5%)
$75,000 or more	15 (17.1%)
Length of time in recovery^‡^	
Less than one month	3 (3.4%)
1–3 months	9 (10.2%)
3 months-6 months	7 (8.0%)
6 months-1 year	13 (14.8%)
1–2 years	10 (11.4%)
3–5 years	14 (15.9%)
More than 5 years	27 (30.7%)

^†Two participants selected “Prefer not to answer” rather than disclose their sex. ‡Three participants endorsed “I am not sure I consider myself as being in recovery” rather than a particular amount of time in recovery.^

### 3.2 Daily meaningfulness

Model results predicting daily meaningfulness are detailed in Table 3. Both greater between-person means of RCC helpful experiences as well as daily RCC helpful experiences were significantly related to higher daily meaningfulness, even when including prior-day autoregressive meaningfulness as a covariate. This means that (1) *individuals* who generally have more helpful experiences at their RCC than their peers are more likely to experience meaningfulness in their day-to-day lives, and (2) on *days* that RCC participants have particularly helpful experiences at their RCC (above their personal average of RCC helpfulness) participants endorse experiencing higher meaningfulness. That the main effects of same-day helpful experiences remained significant when controlling for prior day meaningfulness means that prior day meaningfulness did not account for the observed same-day associations between helpful experiences and meaningfulness.

**Table 3:** Multilevel models predicting daily meaningfulness.

	Only same-day predictors (k = 233)		Including autoregressive predictor (k = 142)	
	Estimate	SE	Estimate	SE
Intercept	12.91	9.10	−0.74	11.09
Personal mean RCC helpfulness	0.77***	0.10	0.84***	0.14
Same-day RCC helpfulness (pmc)	0.61***	0.09	0.68***	0.12
Prior-day meaningfulness			0.10	0.06
Random effects				
Intercept variance	70.43	8.39	43.14	6.57
R^2^	0.34		0.42	

Models allow for random intercepts; k = observed days; pmc = person-mean centered; R^2^ = marginal R^2^; ***p < 0.001.

### 3.3 Daily recovery identity

The results for the models predicting daily recovery identity are provided in Table 4. Between-person means of RCC helpful experiences, as well as daily RCC helpful experiences significantly positively predicted daily meaningfulness, even when including prior-day autoregressive meaningfulness as a covariate. These results indicate that (1) *individuals* who generally have more helpful experiences at their RCC than their peers endorse a strong recovery identity in their day-to-day lives, and (2) on *days* that RCC participants have particularly helpful experiences at their RCC (above their personal average of RCC helpfulness) participants endorse experiencing higher recovery identity than their average over the study. These findings mirror those described in Section 3.2. Similarly, that the main effects of same-day helpful experiences remained significant when controlling for prior day recovery identity means that prior day recovery identity did not account for the observed same-day associations between helpful experiences and recovery identity.

**Table 4:** Multilevel models predicting daily recovery identity.

	Only same-day predictors (*k* = 234)		Including autoregressive predictor (*k* = 142)	
	Estimate	SE	Estimate	SE
Intercept	30.97***	8.51	15.57	8.25
Personal mean RCC helpfulness	0.62***	0.10	0.58***	0.10
Same-day RCC helpfulness (pmc)	0.20**	0.08	0.30**	0.10
Prior-day recovery identity			0.24***	0.06
Random effects				
Intercept variance	85.61	9.25	16.98	4.12
R^2^	0.24		0.45	

Models allow for random intercepts; k = observed days; pmc = person-mean centered; R^2^ = marginal R^2^; **p < 0.01; ***p < 0.001.

## 4 Discussion

RCCs serve as a form of “neighborhood and built environment[s]” (6) which are designed to support mutable indices of recovery. This study explored the daily variability of the helpfulness of RCC experiences, and whether this helpfulness predicted the holistic recovery outcomes of daily meaningfulness and recovery identity. To the best of the authors’ knowledge, this is the first study to quantity daily variability in the quality of RCC experiences.

Nearly half (49%) of the variation in the helpfulness of RCC experiences was attributable to intraindividual variation, or day to day variation. One of the features of RCCs is that they provide opportunities for attendees to engage in many different types of activities and support services. These include formal services such as recovery coaching, and mutual help meetings, as well as informal activities such as socializing with others in recovery. The authors postulate that the intraindividual variation in RCC helpfulness may be due to the different types of activities that attendees participate in from day to day. This study recruited participants from six specific RCCs, and these RCCs have different programming schedules for the various activities they offer. For instance, one of the RCCs hosts recovery support meetings every day that the center is open, while social activities are scheduled a few times a week. Another RCC also hosts meetings every day, but some demographic-specific meetings are only scheduled once or twice a week (for instance, meetings just for women, men, or members of a specific religious group). At all the RCCs, other specific events, such as health or parenting classes may be scheduled only somewhat regularly, and peer recovery coaching sessions may be done as scheduled with one’s peer, and not every time that the RCC is visited. We postulate that different individuals find different activities helpful. For example, a woman in recovery may find a women’s only recovery meeting more helpful than a meeting with both men and women. Interindividual preferences for different activities would then lead to intraindividual variation in helpfulness. The hypothetical woman who prefers women-only meetings would likely rate her visit at the RCC to be more helpful on days when those specific meetings are available, than other visits at the RCC. These day-to-day differences in activities may account for why some days are more helpful than others, even for the same individual.

The *individuals* who reported that their RCC experiences were more helpful on average, relative to their peers, were those who also had greater meaningfulness and a stronger recovery identity in their daily lives. The *days* on which attendees reported that their RCC experiences were particularly helpful, they also reported greater meaningfulness and recovery identity. This was true even when controlling for the prior-day outcome, implying that RCC helpfulness predicts higher indices of recovery well-being, accounting for the prior day’s potential effects on these associations. These findings suggest that RCCs are meeting their objective to support multiple aspects of recovery both in the current day, as well as cumulatively across days.

These findings both confirm, and extend on, prior cross-sectional findings that RCC attendance was associated with positive recovery outcomes (13). This study confirms these findings by offering additional evidence that RCCs are associated with indices of holistic recovery, with improved ecological validity by utilizing a daily diary approach. The current study extends on the prior findings by assessing different indices of recovery (meaningfulness and recovery identity vs. psychological distress, self-esteem and quality of life), illustrating that the helpfulness of RCC experiences varies from visit to visit, and that differences in helpfulness are associated with differences in indices of recovery, in the day to day lives of people in recovery.

These findings should be viewed in light of important limitations. The first is that only six RCCs, and 88 attendees, in a geographically constrained area, were included in the study. These limited numbers of RCCs and attendees are likely not generalizable to RCCs at large. The RCCs were almost all located in industrial, semi-rural towns, and all the RCCs were in the state of Pennsylvania. The findings described herein are not likely to generalize to RCC attendees in urban areas, or outside of the Northeastern United States. Future studies should consider recruiting RCC attendees from urban, suburban, semi-rural and rural areas, from various regions in the United States, as well as recruiting more participants, as funding allows.

Additionally, this study utilized a sampling approach that likely influenced the findings. We suspect that those who self-selected to attend the recruitment meetings and participate in a study about recovery are also likely to be those who have high recovery identities and are particularly committed to their recovery in general. This assumption is supported by the fact that nearly half of the sample had been in recovery for 3 years or longer. The impact of these sampling issues–which are not uncommon in recovery and treatment research—and their implications for generalizability should be kept in mind when considering findings. Finally, because RCC helpfulness (the predictor) and the indices of recovery (the outcomes) were measured on the same day, causality cannot be inferred. It is possible that participants who were already having a good recovery day, such as a day with high recovery identity, or meaningfulness, will have been more engaged during their RCC visit, and reported that visit to be particularly helpful. This is in contrast with the explanation that RCC helpfulness *promotes* daily holistic indices of recovery. Future studies could assess the cross-lagged relationship between RCC experiences, and recovery outcomes, from one day to the next. Future research could also assess other holistic indices of recovery which may be related to RCCs, such as positive affect, and positive social interactions.

## 5 Conclusion

RCCs are an important resource for those who need flexible access to diverse forms of recovery support. RCCs predict the holistic recovery outcomes of meaningfulness and recovery identity outcomes on the particular days that the RCCs are visited, and for the individuals who find RCCs more helpful overall. While more research is needed on which aspects of RCC attendance are particularly helpful and for whom, these findings suggest that RCC engagement may be a useful component of future just-in-time interventions, intended to support recovery at times when it is particularly needed. This study also offers preliminary evidence to suggest that RCCs are appropriate recipients of public funding intended to support recovery in US communities, such as those funds recently received by US states through settlements with opioid pharmaceutical companies.

## Data Availability

The raw data supporting the conclusions of this article will be made available by the authors, without undue reservation.
