# Supplementary material for: Experiences at recovery community centers predict holistic recovery outcomes: a daily diary assessment of RCC helpfulness, meaningfulness, and recovery identity
Source: Front Public Health. 2025 Jan 14;12:1476441. doi: 10.3389/fpubh.2024.1476441 (PMC11772195; doi:10.3389/fpubh.2024.1476441)
Supplement: Supplementary file 1 [file Table_1.docx]

| Supplemental Table 1. Items in the daily scales | | | |
| --- | --- | --- | --- |
| RCC Quality Items | | | |
| **Item** | **M** | **SD** | **Range** |
| How helpful to your recovery was being at the RCC? | 85.97 | 16.61 | 21-100 |
| How helpful to your overall well-being was being at the RCC? | 86.06 | 17.1 | 21-100 |
| How supported did you feel by other members and staff at the RCC? | 88.13 | 16.85 | 15-100 |
| How connected did you feel to other members and staff at the RCC? | 87.75 | 16.85 | 15-100 |
| How accepted did you feel by other members and staff at the RCC? | 88.65 | 16.04 | 9-100 |
| How helpful were the support/resources provided by the RCC? | 87.73 | 16.18 | 28-100 |
| How helpful was any information you got at the RCC? | 86.78 | 16.23 | 28-100 |
| RCC Recovery Identity Items; *Thinking about today, I feel like…* | | | |
| **Item** | **M** | **SD** | **Range** |
| I was committed to my recovery | 87.4 | 18.01 | 0-100 |
| I worked hard on my recovery | 81.88 | 21.83 | 0-100 |
| I kept my recovery central to my day | 83.09 | 19.38 | 0-100 |
| I felt like a "person in recovery" | 86.45 | 18.84 | 0-100 |
| I felt connected to other people in recovery | 86.15 | 17.94 | 16-100 |
| I was grateful to be in recovery | 89.67 | 15.46 | 0-100 |
| I thought of myself as being part of the recovery community, even when I was not with other people in recovery | 86.47 | 17.99 | 20-100 |
| My being in recovery guided my decisions | 85.86 | 17.53 | 0-100 |
| I missed my old drug use/drinking social group | 83.34 | 26.26 | 0-100 |
